# Elucidation of the binding mode of organic polysulfides on the human TRPA1 receptor

**DOI:** 10.3389/fphys.2023.1180896

**Published:** 2023-06-07

**Authors:** Balázs Nemes, Szabolcs László, Balázs Zoltán Zsidó, Csaba Hetényi, Adam Feher, Ferenc Papp, Zoltan Varga, Éva Szőke, Zoltán Sándor, Erika Pintér

**Affiliations:** ^1^ Department of Pharmacology and Pharmacotherapy, Medical School, University of Pécs, Pécs, Hungary; ^2^ Department of Inorganic and Analytical Chemistry, Faculty of Chemical Technology and Biotechnology, Budapest University of Technology and Economics, Budapest, Hungary; ^3^ Department of Biophysics and Cell Biology, Faculty of Medicine, University of Debrecen, Debrecen, Hungary

**Keywords:** human TRPA1, site-directed mutagenesis, DMTS, DATS, DADS, binding site, organic polysulfide, electrophilic TRPA1 agonist

## Abstract

**Introduction:** Previous studies have established that endogenous inorganic polysulfides have significant biological actions activating the Transient Receptor Potential Ankyrin 1 (TRPA1) receptor. Organic polysulfides exert similar effects, but they are much more stable molecules, therefore these compounds are more suitable as drugs. In this study, we aimed to better understand the mechanism of action of organic polysulfides by identification of their binding site on the TRPA1 receptor.

**Methods:** Polysulfides can readily interact with the thiol side chain of the cysteine residues of the protein. To investigate their role in the TRPA1 activation, we replaced several cysteine residues by alanine via site-directed mutagenesis. We searched for TRPA1 mutant variants with decreased or lost activating effect of the polysulfides, but with other functions remaining intact (such as the effects of non-electrophilic agonists and antagonists). The binding properties of the mutant receptors were analyzed by *in silico* molecular docking. Functional changes were tested by *in vitro* methods: calcium sensitive fluorescent flow cytometry, whole-cell patch-clamp and radioactive calcium-45 liquid scintillation counting.

**Results:** The cysteines forming the conventional binding site of electrophilic agonists, namely C621, C641 and C665 also bind the organic polysulfides, with the key role of C621. However, only their combined mutation abolished completely the organic polysulfide-induced activation of the receptor.

**Discussion:** Since previous papers provided evidence that organic polysulfides exert analgesic and anti-inflammatory actions in different *in vivo* animal models, we anticipate that the development of TRPA1-targeted, organic polysulfide-based drugs will be promoted by this identification of the binding site.

## 1 Introduction

Chronic pain and persistent inflammation are major problems in the modern population (Mha et al., 2008; Apkarian et al., 2009; Harden et al., 2010; Nathan and Ding, 2010). Chronic pain is a direct cause of diminishing quality of life or even loss of body functions (Niv and Kreitler, 2001; Keeley et al., 2008; Helyes et al., 2019; Raja et al., 2020). Persistent inflammation without pain sensation may stay latent, but can contribute to a wide variety of other chronic diseases, such as type II diabetes, allergies, cardiovascular diseases, and several types of cancer (Medzhitov, 2021; Pahwa et al., 2021; Nathan, 2022). Conventional analgesic and anti-inflammatory drugs, such as steroids, NSAIDs, and opioids are, commonly unsuitable for long-term treatments because of their gradually overwhelming adverse effects (Alarcón, 2000; Schett and Gravallese, 2012; Schuelert and McDougall, 2012; Horváth et al., 2016; Cavalli et al., 2019). Therefore, there is a great demand for new drugs against chronic pain and persistent inflammation. Polysulfides could be promising drug candidates and became increasingly well-studied compounds in pharmacological research. Formerly, the gaseous regulator hydrogen sulfide (H_2_S) was thought to have analgesic and anti-inflammatory effects. Now, it is well-understood that H_2_S released in the location of inflammation is spontaneously oxidized into sodium hydrosulfide (NaSH) and sodium sulfide (Na_2_S) and polymerized into inorganic polysulfides (e.g. Na_2_S_3_). These compounds are reactive enough to bind covalently to the cysteine residues of the transient receptor potential ankyrin 1 (TRPA1) receptor and activate it (Ogawa et al., 2012; Hatakeyama et al., 2015; Pozsgai et al., 2017; Bátai et al., 2019).

TRPA1 is a non-selective cation channel sensing a wide variety of irritations such as mechanical stimuli, extreme heat and cold, acids, reactive oxidative species, and thousands of identified agonists (Liu et al., 2021). TRPA1 is mainly expressed in the capsaicin-sensitive peptidergic nociceptive primary sensory neurons; therefore, receptor activation leads to local acute pain and to the secretion of pro-inflammatory neuropeptides (e.g., substance P and calcitonin gene-related peptide), which contribute to neurogenic inflammation by prompting vasodilation and plasma leakage. However, as a counter-regulation, these peptidergic neurons also release analgesic and anti-inflammatory neuropeptides such as somatostatin upon TRPA1 activation (Kun et al., 2014; Zygmunt and Högestätt, 2014; Pozsgai et al., 2017; Dombi et al., 2021). Somatostatin has systemic analgesic and anti-inflammatory effects mediated by the SST_4_ receptor (Matsuoka et al., 1994; Helyes et al., 2006; 2009; Sándor et al., 2006; Qiu et al., 2008; Markovics et al., 2012; Pintér et al., 2014; Scheich et al., 2016; 2017; Nemes et al., 2021). Gene knockout in mice of either *Trpa1* or *Sstr4* significantly attenuates these effects of polysulfides (Pozsgai et al., 2017). Based on the previous data listed previously, we assume that polysulfides exert their anti-inflammatory and pain-relieving effects, at least in part, through the activation of the TRPA1 receptor.

Despite the advantageous effects of inorganic polysulfides, they are highly unstable molecules; therefore, their delivery to the target cells or endogenous synthesis from added H_2_S donors is extremely challenging, which makes them unsuitable as drugs. So, our attention turned to the much more stable organic polysulfides such as dimethyl trisulfide (DMTS), diallyl disulfide (DADS), and diallyl trisulfide (DATS), which have similar biological effects (Bai et al., 2005; Bautista et al., 2005; Koizumi et al., 2009; Lee et al., 2013; Pozsgai et al., 2017; Bátai et al., 2018; Dombi et al., 2021).

**FIGURE 1:**
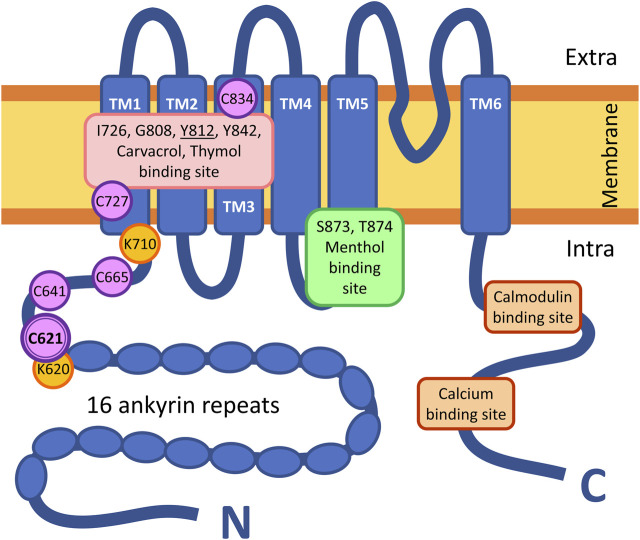
Structure of a subunit of the homotetrameric TRPA1 receptor. TM = transmembrane domain; intra = intracellular space; extra = extracellular space; N = N-terminus of the peptide, C = C-terminus of the peptide. Symbols of the highlighted amino acids: C = cysteine; G = glutamine; I = isoleucine; K = lysine; S = serine; T = threonine; Y = tyrosine.

Although the molecular mechanism of action of inorganic polysulfides has been better studied, little is known about organic polysulfides. In the first step, we aimed to identify the binding site of organic polysulfides on the TRPA1 receptor via site-directed mutagenesis. Therefore, out of the 28 cysteine residues in the human TRPA1, we investigated those within the conventional binding site of electrophilic agonists on the N-terminal domain (C621, C641, and C665) (Hinman et al., 2006; Deering-Rice et al., 2011; Eberhardt et al., 2012; Shapiro et al., 2013) and the theoretical binding site of the strongly hydrophobic agonists in the transmembrane domains (C727 and C834) (Figure 1) (Paulsen et al., 2015; Alvarado et al., 2021). We created mutant TRPA1 variants, which lost the sensitivity to the tested organic polysulfides, but their other functions remained intact (e.g., sensitivity to non-electrophilic agonists and antagonists). The binding properties were calculated *in silico*, and the receptor functions were tested *in vitro* by calcium-sensitive fluorescent flow cytometry, radioactive calcium-45 liquid scintillation counting, and whole-cell patch clamp.

## 2 Materials and methods

### 2.1 Computational docking of DMTS, DADS, and DATS

Dimethyl trisulfide (DMTS), diallyl disulfide (DADS), and diallyl trisulfide (DATS) were prepared by the Sketcher 2D tool of FITTED, and the CONVERT tool performed the conversion into a mol2 file. The ligands were further prepared by the SMART tool of FITTED (Corbeil et al., 2007; Therrien et al., 2012; Pottel et al., 2014). The torsion around all rotatable bonds was allowed, AMBER (Wang et al., 2004) atom types were assigned, and Gasteiger–Hückel (Gasteiger and Marsili, 1980) partial charges were added. The same ligands were prepared for AutoDock (RRID:SCR_012746 (Morris et al., 1998)) calculations, as described before (Mohos et al., 2020a; 2020b; Zsidó et al., 2020; Fliszár-Nyúl et al., 2021); briefly, the structures were built by Maestro (Schrödinger, 2022), energy was minimized by OpenBabel (RRID: SCR_014920), and Gasteiger–Marsili partial charges were added with OpenBabel (O’Boyle et al., 2011).

The atomic coordinate file of the ligand-free TRPA1 receptor was obtained from the Worldwide Protein Data Bank (wwPDB, RRID:SCR_006555 (Berman et al., 2002)), under the accession code 6V9W (Zhao et al., 2020). As the four chains of the target are symmetrical (homotetramer), only one chain was used to reduce computational costs. The amino acids of a chain do not interfere with the binding of the ligand to another chain. The missing atoms and residues were rebuilt using SWISS-MODEL (Waterhouse et al., 2018), and energy was minimized with GROMACS (Van Der Spoel et al., 2005). The convergence threshold of the steepest descent optimization was set to 103 kJ mol−1 nm−1, and that of the conjugate gradient optimization to 10 kJ mol−1 nm−1. The AMBER99SB-ILDN force field (Wang et al., 2004) was used for calculation, and a position restraint at a force constant of 103 kJ mol-1 nm-2 was applied on heavy atoms. The same preparation process was carried out for the mutant (C621S) receptor, accessible under the PDB code 6PQQ (Suo et al., 2020), and the holo (6PQP) receptor. The holo receptor contains benzyl isothiocyanate (BITC) covalently bound to the C621 amino acid, BITC was removed, and the hydrogen atom was restored on the target side before calculations. The targets were further optimized by the ProCESS tool of FITTED, with the original settings (Corbeil et al., 2007). In the case of AutoDock, the added H atoms and partial charges were kept from energy minimization. The mutation of the 6PQQ apo receptor S621A was performed by PyMol (The PyMOL Molecular Graphics System, 2022).

Covalent docking calculations were carried out using FITTED. The covalent residue (C621 or S621) and adjacent basic residue (P622) were adjusted in the graphical user interface of the program. Root mean square deviation (RMSD) values were calculated between the crystallographic and representative ligand conformations, if available. All other settings were used as the default of the program. Ten docking runs were performed for all ligands, and the resulting ligand conformations were ranked based on their calculated free energy of binding (ΔG_calc_) values.

### 2.2 Organic polysulfide synthesis

#### 2.2.1 Chemicals

Dimethyl trisulfide, sulfur, sodium sulfide, ammonium hydroxide, ethanol, and activated charcoal were purchased from Reanal, Hungary. Cysteine (168149), tetrabutyl ammonium iodide (140775), and allyl bromide (337528) were purchased from Sigma-Aldrich.

All reagents and materials were used without purification.

#### 2.2.2 Instruments

For the synthesis of the two sulfides we prepared, we used a modified version of the method found in the literature (Yuan et al., 2006). In our reaction, by using a more efficient phase transfer catalyst than the published one, the reaction could be carried out in a more energy-efficient way and with simpler tools, while maintaining the original production and purity. Infrared spectra were recorded on a PerkinElmer Spectrum Two Fourier-transform infrared spectrometer (FTIR) with a universal attenuated total reflectance accessory (UATR) head. Mass spectra (MS) and the analysis of the product compounds were obtained by a GC/MS QP-2010 spectrometer (EI, 70 eV). The capillary column used was ZB5-MSI, 30 m in length and 0.25 mm in diameter. The conditions were as follows: column temperature was set at 80°C and then increased to 250°C; helium was used as carrier gas at a linear flow of 1 ml/min.

#### 2.2.3 Diallyl disulfide and diallyl trisulfide

Both compounds were prepared by a modified version of the method published by Yuan et al. (2006). In the first step, sodium disulfide and sodium trisulfide solutions were prepared by sulfur and sodium sulfide as follows: for diallyl disulfide: 6.4 g (0.2 mol) sulfur and 48 g (0.2 mol) sodium sulfide, and for diallyl trisulfide: 6.4 g (0.2 mol) sulfur and 24 g (0.1 mol) sodium sulfide, and the solution was dissolved in 100 ml distilled water in a round-bottom flask. The solutions were stirred at RT for 1 day and then filtrated using a paper filter. About 0.3 g tetrabutyl-ammonium iodide was added to the brownish red solutions as a phase transfer catalyst. For diallyl disulfide and diallyl trisulfide, 36.3 g (0.3 mol) allyl bromide and 18.2 g (0.15 mol) allyl bromide were added dropwise in 20 min, respectively, and the temperature of the reaction mixture was increased. After addition, the reaction mixtures were cooled with ice. The resulting mixtures were extracted with 300 ml ether. The organic phase was dried over anhydrous MgSO_4_ overnight. The organic phase was placed on a rotary evaporator under aspirator pressure to eliminate the solvent. Then, the residual phase was distilled in low vacuum.

Diallyl disulfide: the fraction at 45°C–60°C and 0.8 torr. was obtained. In addition, 25.8 g diallyl disulfide oil with light yellow color and intensive garlic smell was obtained with the yield of 88%, purity (GC-MS): 90% (Supplementary Figure S1).

The functional group was identified by FT-IR (UATR, cm-1): 3,081 (=C-H); 3,009 (-CH2); 2,979 (-CH2); 1,634 (C=C); 1,422 (C-S). (Supplementary Figure S2).

Diallyl trisulfide: the fraction at 90°C–110°C and 1.0 torr. was obtained . In addition, 5.1 g diallyl trisulfide oil with yellow color and intensive garlic smell was obtained with the yield of 37%, purity (GC-MS): 63.5% (Supplementary Figure S3).

The functional group was identified by FT-IR (UATR, cm-1): 3,081 (=C-H); 3,009 (-CH2); 2,979 (-CH2); 1,634 (C=C); 1,422 (C-S). (Supplementary Figure S4).

The impurity of DADS was DATS, and the impurity of DATS was DADS. No other contaminant was found in either compound. The difference in retention times for GC-MS measurements is due to instrument maintenance between the two measurements (Supplementary Figures S1, 3).

### 2.3 Site-directed mutagenesis via PCR

Our research group has already created an expression vector containing the wild-type human TRPA1 cDNA, which is now called pT31-hTRPA1, and its creation was described by Pozsgai et al. (2017). This plasmid contains the TRPA1 coding sequence within a Sleeping Beauty 100 transposon, flanked by a cytomegalovirus (CMV) promoter at the 5′ end and the bovine growth hormone polyA at the 3’ end. Selective markers were ampicillin, kanamycin/neomycin, and geneticin (G418) resistance genes. Although this vector was originally prepared for integrating the transgene into the chromosome of the CHO cells, we only used it for transient transfection, without the SB100 transposase enzyme.

**FIGURE 2:**
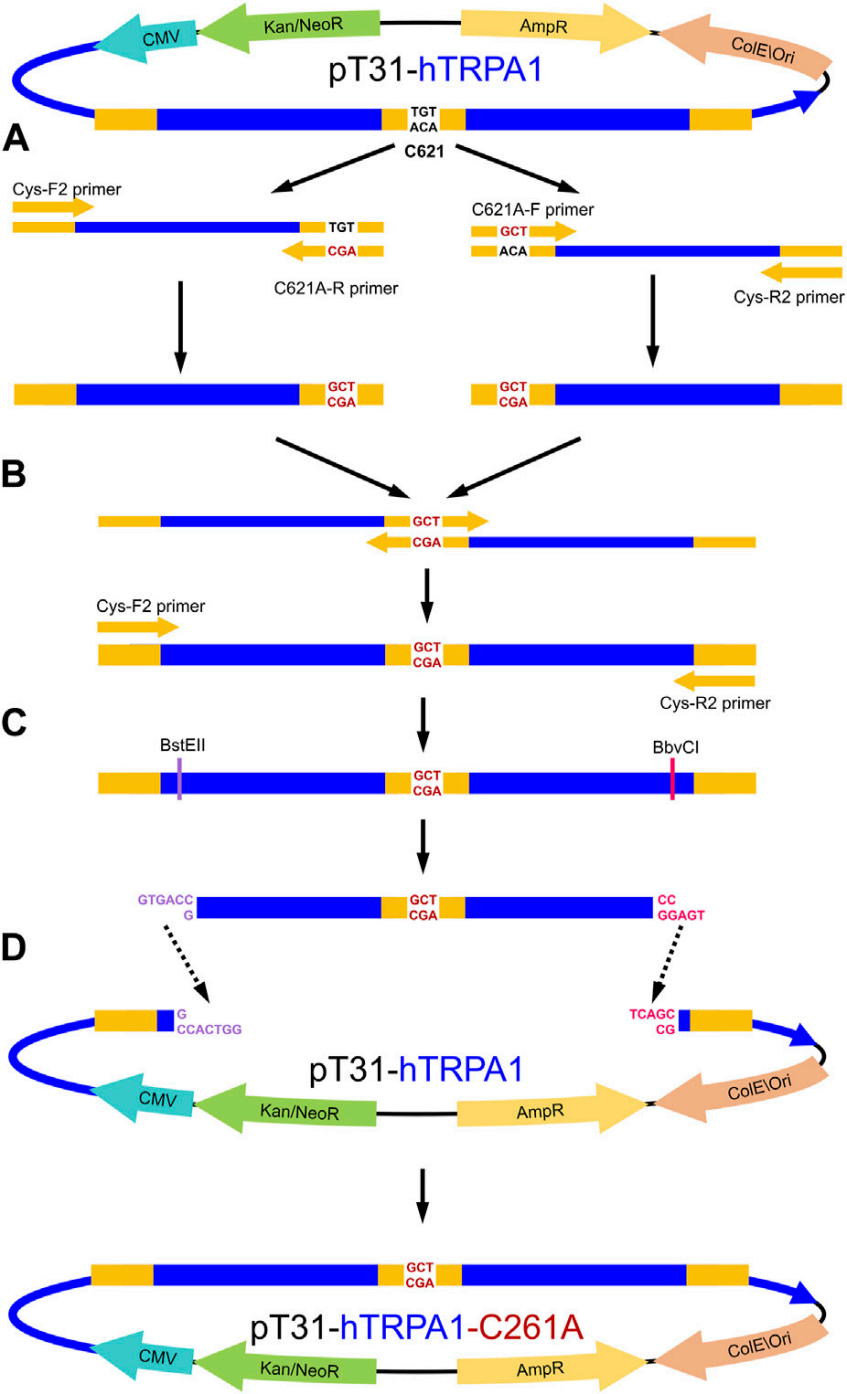
Process of the *TRPA1* PCR-based site-directed mutagenesis, with the example of C621A mutation. **(A)** Starting from the wild-type *TRPA1* coding pT31-hTRPA1 plasmid, in the first PCR, the two halves of the cloning region were separately amplified by a mixed pair of mutation primer and cloning primer (in this case: Cys-F2 with C621A-R, and C621A-F with Cys-R2). **(B)** In the second PCR, the two halves were joined and amplified by Cys-F2 and Cys-R2 cloning primers. **(C)** Mutation-carrying complete PCR product and the original pT31-hTRPA1 plasmid were sequentially digested by BstEII and BbvCI unique restriction endonucleases. **(D)** Mutation-carrying PCR product was ligated into the “empty” plasmid, creating the mutant *TRPA1* coding expression vector. CMV–cytomegalovirus promoter; AmpR–ampicillin resistance cassette; Kan/NeoR–kanamycin and neomycin resistance cassette; ColE\Ori–*E. coli* specific origin of replication; blue region–TRPA1 cDNA; orange–primer sites; black-lettered bases–codon of the 621st cysteine; red-lettered bases–codon of alanine (C621A mutation); purple-lettered bases–DNA sticky end after BstEII cleaving; pink-lettered bases–DNA sticky end after BbvCI cleaving.

The single mutant TRPA1 variants were created in two consecutive PCR reactions (Supplementary Tables S1A, B). TAdvanced Twin (Analytik Jena 846-2-070-2xx) PCR machine and Q5 Hot Start High-Fidelity DNA polymerase (New England Biolabs M0493) were used for all PCR reactions, following the manufacturer’s manuals. All primers were designed by Primer-BLAST (RRID: SCR_003095 (Ye et al., 2012)) and Integrated DNA Technologies (IDT) OligoAnalyzer Tool (RRID:SCR_001363 (Owczarzy et al., 2008)), synthesized by IDT. Mutation-carrying primers were designed for each codon of the targeted amino acids (C621, C641, C665, C727, and C834), both in forward and reverse orientation (Supplementary Table S2). Terminal primers CysF2 and CysR2 (Supplementary Table S2) were designed to include unique restriction endonuclease cutting sites (BstEII near the 5′ end and BbvCI near the 3′ end) in the PCR product. In the first PCR (Supplementary Table S1A), the mutation-carrying primers were paired with their opposing terminal primer, each orientation in a separate reaction (e.g., CysF2 and C621A-R; C621A-F and CysR2) creating two halves of the intended final mutation-carrying PCR product (Figure 2A). These halves were combined in the second PCR by the terminal primers (Figure 2B; Supplementary Table S1B). The final PCR products and intact pT31-hTRPA1 plasmid were digested by BstEII-HF (New England Biolabs R3162) and BbvCI (New England Biolabs R0601) enzymes (Figure 2C), then isolated by agarose gel electrophoresis, and extracted by NucleoSpin DNA Gel and PCR Clean-up kit (Macherey-Nagel 740609.50). The vector fragment without the intact TRPA1 coding fragment (8,192 bp) and the new mutation-carrying fragment (1,461 bp) were ligated by T4 DNA ligase (New England Biolabs M0202L) (Figure 2D).

Double- and triple-mutant TRPA1 variants were created the same way, except they were produced from single- and double-mutant pT31-hTRPA1 variants, respectively, rather than from the WT. All TRPA1 mutant variants were examined by Cys-F and Cys-R sequencing primers in Sanger sequencing (Szentágothai Research Centre, University of Pécs, Hungary) for carrying the intended mutations but lacking any unintended mutation.

### 2.4 Plasmid amplification in *E. coli*

Chemically competent DH5α *E. coli* cells were prepared by the calcium chloride method (Chang et al., 2017). About 10 µl ligated vector was added to 100 µl competent cells, and then the cells were incubated for 30 min on ice. The cells were transformed by heat-shock (42 °C for 60 s), then 1 ml LB medium was added, and the cells were incubated at 37 °C for 1 h. Then, 200 μl cells was spread on 100 μg/ml ampicillin (Amp) selective LB-agar plates and incubated at 37°C overnight. The grown colonies were streaked on new Amp plates (max. six colonies per TRPA1 mutant variant) and incubated at 37 °C overnight. One colony of each cell line was transferred to 1 ml 100 μg/ml ampicillin selective LB medium and incubated with continuous shaking at 200 rpm at 37 °C overnight. The plasmids were isolated by the GeneJET Plasmid Miniprep Kit (Thermo Fisher Scientific K0503). The plasmid concentration was measured by the Jenway 7310 UV/Vis spectrophotometer. First, the plasmids were tested by a diagnostic digest by SpeI (R0133), PvuI (R0150), PstI (R0140), and EcoRI (R0101) DNA restriction endonucleases from New England Biolabs. Second, intact plasmids were tested by Sanger sequencing (Bioinformatical Research Group, Szentágothai Research Centre, University of Pécs) using the CysF and CysR primers (Supplementary Table S2). The raw sequencing data were analyzed by the ApE A plasmid Editor (RRID: SCR_014266) and Nucleotide-BLAST (NIH, NCBI RRID: SCR_001598) and compared to those of the TRPA1 WT in pT31-hTRPA1 plasmid. Only those plasmids were suitable and used for later research, which contained only the intended mutation. These selected plasmids were amplified by transferring the carrying bacterium cells from the streaking plate to 200 ml and 100 μg/ml ampicillin selective LB medium, and these cells were incubated with continuous shaking at 200 rpm at 37 °C overnight. A high amount of plasmid DNA was isolated by the NucleoBond Xtra Maxi Plus EF Kit (Macherey-Nagel 740426.50), and their concentration was measured by the Jenway 7310 UV/Vis spectrophotometer. The final concentration of each plasmid was set to 250 ng/μl.

### 2.5 CHO transfection

The Chinese hamster ovary (CHO, RRID: CVCL_0213) cells were transfected by TurboFect (Thermo Fisher Scientific R0532). Regarding the TurboFect–DNA–medium ratio, some alterations from the manufacturer’s recommendation were needed to achieve an optimal transfection rate. The empirical data showed best results with 10 µl TurboFect and 10 µl plasmid (2.5 µg DNA) for 1 ml CHO cells at 50% confluency in a 24-well plate (∼100,000 cells per well) instead of 2 µl TurboFect and 1 µg plasmid recommended by the manufacturer. TurboFect and the plasmid were mixed in 90 µl Opti-MEM (Thermo Fisher Scientific 31985062) and were incubated at room temperature (RT) for 30 min before adding to 1 ml CHO cells. The CHO cells were incubated at 37 °C for 24 h before the experiment. Since this method is a transient transfection, the TRPA1 expression was drastically decreased after 48 h, and the cells became unsuitable for experimentation.

The expression of TRPA1 variants was tested by immunohistochemistry, using polyclonal anti-human TRPA1 rabbit IgG primary antibody (Thermo Fisher OST00061W, RRID: AB_2207890) and peroxidase-linked polyclonal anti-rabbit IgG goat IgG secondary antibody (Thermo Fisher Scientific 31460, RRID: AB_228341), and stained by the 3,3′-diaminobenzidine (DAB) Liquid Substrate System (Sigma-Aldrich D7304). Non-transfected CHO cells were used as negative control. The resulting microscopic images were measured by pixel darkness analysis. Immunohistochemistry showed no inhibition in the TRPA1 expression due to the mutations (data not shown).

### 2.6. Fluo-4 flow cytometry

In total, 1 ml (∼100,000 cells) of each transfected CHO cell line was stained by 4 µl Invitrogen Fluo-4, AM (Invitrogen, Thermo Fisher Scientific F14201) cell permeant calcium indicator fluorescent dye at 37 °C for 1 h in non-adhesive tubes. For each sample condition, 100 µl of CHO cells was transferred to a fresh tube and 900 µl reagent was added. The reagents were organic polysulfides (DMTS, 90% DADS, 63.5% DATS), other electrophilic agonists (allyl isothiocyanate (AITC) and cinnamaldehyde, JT010), non-electrophilic agonists (carvacrol, menthol, and thymol), and antagonists (HC-030031 and A-967079). The vehicle and control solutions were the extracellular solution (ECS) buffer containing 1 mM CaCl_2_. The final concentration of reagents was mostly 100 μM, except for the much more competent JT010, which needed to be used always in 1,000 x dilution compared to other agonists (usually in 100 nM).

Right after adding the agonist, each sample was measured for green fluorescence (excitation: 488 nm, emission: 516 nm, filter: 527/30) by the CyFlow Space Flow Cytometer (Sysmex Partec, Germany). The results were analyzed by FloMax (RRID: SCR_014437) and GraphPad Prism 8 (RRID: SCR_002798) software. The data were analyzed by the following equation:

$$relative\;TRPA1\;activating\;effect=\frac{I_{electrophilic\;agonist}-I_{control}}{I_{Carvacrol}-I_{control}},$$

where I is the average fluorescence (512–542 nm) intensity of the cells per sample. Electrophilic agonists were the DMTS, 63.5% DATS, 90% DADS, AITC, and JT010. ECS buffer was the control.

### 2.7 Radioactive calcium-45 uptake experiments in CHO cells expressing mutant TRPA1 receptor variants

Transiently transfected CHO cells expressing TRPA1 WT, C621A, C641A, C665A, and C621A/C641A/C665A mutants were grown to approximately 70% confluency in a 24-well plate, then digested in trypsin for 5 min, and suspended in 300 µl complete DMEM per well. The suspended cells were transferred to 72-well microbatch plates (15 µl suspended cells per well) and incubated overnight to adhere properly to the bottom of the well. The following day, the cells were washed five times with calcium-free Hank’s solution (pH 7.4) and incubated in 10 μL of the same buffer containing 200 μCi/ml ^45^Ca isotope (1.3 Ci/mmol, PerkinElmer NEZ013001MC) and 100 µM of either carvacrol (Sigma-Aldrich 282197), AITC (Sigma-Aldrich 377430), DMTS (Sigma-Aldrich W327506), 63.5% DATS, or 90% DADS (and control sample without the TRPA1 agonist) for 2 min at room temperature. After washing five times with ice-cold ECS, the residual buffer was evaporated in 10 min at 37°C. Then, the retained isotope in the cells was collected in 15 μL of 0.1% SDS, and the radioactivity was measured in 2 ml Ultima Gold XR scintillation liquid (Packard BioScience 6013119) in a Packard Tri-Carb 2800 TR scintillation counter. The data were analyzed by the following equation:

$$relative\;TRPA1\;activating\;effect=\frac{{CPM}_{electrophilic\;agonist}-{CPM}_{control}}{{CPM}_{Carvacrol}-{CPM}_{control}},$$

where CPM means “count per minute”, the number of radioactive decays per sample measured during 2 min and averaged for 1 min. The electrophilic agonists were the DMTS, 63.5% DATS, 90% DADS, and AITC. The calcium-free Hank’s solution was the control.

### 2.8 Whole-cell patch clamp

Whole-cell currents of voltage-clamped cells were recorded through manual patch-clamp electrophysiology according to standard protocols using Axopatch 200B amplifiers connected to a computer and digitized with Digidata 1550B (Molecular Devices, San Jose, CA, United States). Data were acquired with pClamp10.7 (Molecular Devices, San Jose, CA, United States). In general, currents were low-pass-filtered using the built-in analog four-pole Bessel filters of the amplifiers and sampled at 5 kHz. Before analysis, whole-cell current traces were digitally filtered (five-point boxcar smoothing). GFP-positive TRPA1 co-transfected CHO (Chinese hamster ovary) cells were identified using a Nikon Eclipse TE2000-U fluorescence microscope (Auro-science LLC, Budapest, Hungary). Pipettes were pulled from GC 150F-15 borosilicate glass capillaries (Harvard Apparatus, Holliston, MA, United States) in five stages with 4–10 MΩ resistance. Before the measurement, the cells were maintained in the recording Petri dish in a bath solution that consisted of 145 mM NaCl, 5 mM KCl, 1 mM MgCl_2_, 2.5 mM CaCl_2_, 5.5 mM glucose, and 10 mM HEPES (pH 7.35; 302–308 mOsmol/kg). For the recordings, the composition of the solution used in the patch pipette (internal solution) was 150 mM NaCl, 10 mM HEPES, and 2 mM EDTA-Na (Ca^2+^-free solution; pH 7.35; ∼300 mOsmol/kg). The composition of the external (control) solution was the same as that of the pipette solution, with the addition of 1 V/V% DMSO as carvacrol was dissolved in DMSO. The solution exchange was achieved by a gravity-driven perfusion system with continuous excess fluid removal. Peak currents were measured every 5 s during 20 m steps at +50 mV after 100-m voltage ramps from 0 mV to +50 mV using a holding potential of 0 mV. To quantify the current activating effect of the activators, if the current increase was detected within 100 s, we waited for its saturation, defined by no further current increase in a 20-s-long period. Only measurements where the positive control agonist (100 µM carvacrol) and antagonist (50 µM HC-030031) showed the desired effect were included in the analysis.

#### 2.8.1 Data analysis

Clampfit 10.7 (Molecular Devices, San Jose, CA, United States of America) and GraphPad Prism 7 (Graphpad, San Diego, CA, United States of America) were used for data display and analysis. The current activation ratio was quantified by the following equation:

$$\frac{I_{DMTS}}{I_{Carvacrol}}=\frac{I_{DMTS\;peak}-I_{control}}{I_{Carvacrol\;peak}-I_{control}},$$

where I_DMTS_ and I_Carvacrol_ are the activated TRPA1 currents by DMTS and carvacrol, respectively. The control current (I_control_) is the peak current measured before the addition of the DMTS, I_DMTS peak_ is the peak current measured in the presence of 100 µM DMTS, and I_Carvacrol peak_ is the peak current measured in the presence of 100 µM carvacrol, a known activator of TRPA1. This calculation was used for the other polysulfides (90% DADS, 63.5% DATS in 100 µM) as well.

To investigate whether point mutations altered the response to the application of the tested agonists, we used one-way ANOVA with Dunnett’s multiple comparison post-hoc test. Differences were labeled as significant if *p* < 0.05.

## 3 Results

### 3.1 Computational docking of DMTS, DADS, and DATS

The ligand-binding conformation of DMTS, DADS, and DATS was calculated targeting the ligand-free conformation of the TRPA1 receptor (6V9W) as described in the Methods section. The docking calculations were also performed on two mutant receptors (6PQQ: C621S and a modeled C621A receptor) and the holo receptor (6PQP) to analyze the effect of the mutation on ligand binding at an atomic resolution level.

**FIGURE 3:**
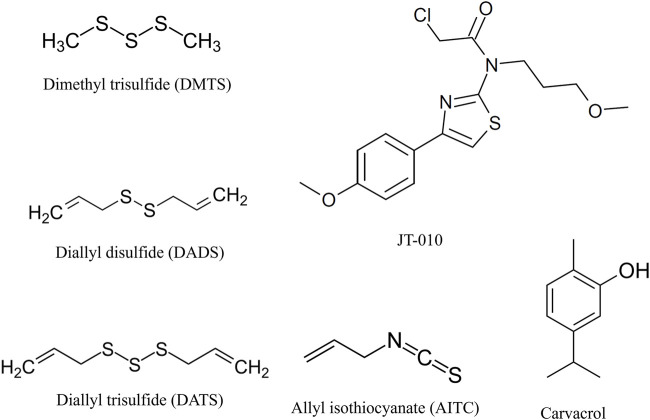
Structure of tested TRPA1 agonists. Organic polysulfides, dimethyl trisulfide (DMTS), diallyl trisulfide (DATS), and diallyl disulfide (DADS), are reversible electrophile agonists. JT010 is a very potent and selective irreversible electrophile TRPA1 agonist. Allyl isothiocyanate (AITC) or mustard oil is a potent natural reversible electrophile TRPA1 agonist. Carvacrol is a non-electrophile TRPA1 agonist with different binding sites on TRPA1, and we used it as a control activator. Illustration is made by ChemDraw Direct (RRID: SCR_016768).

**FIGURE 4:**
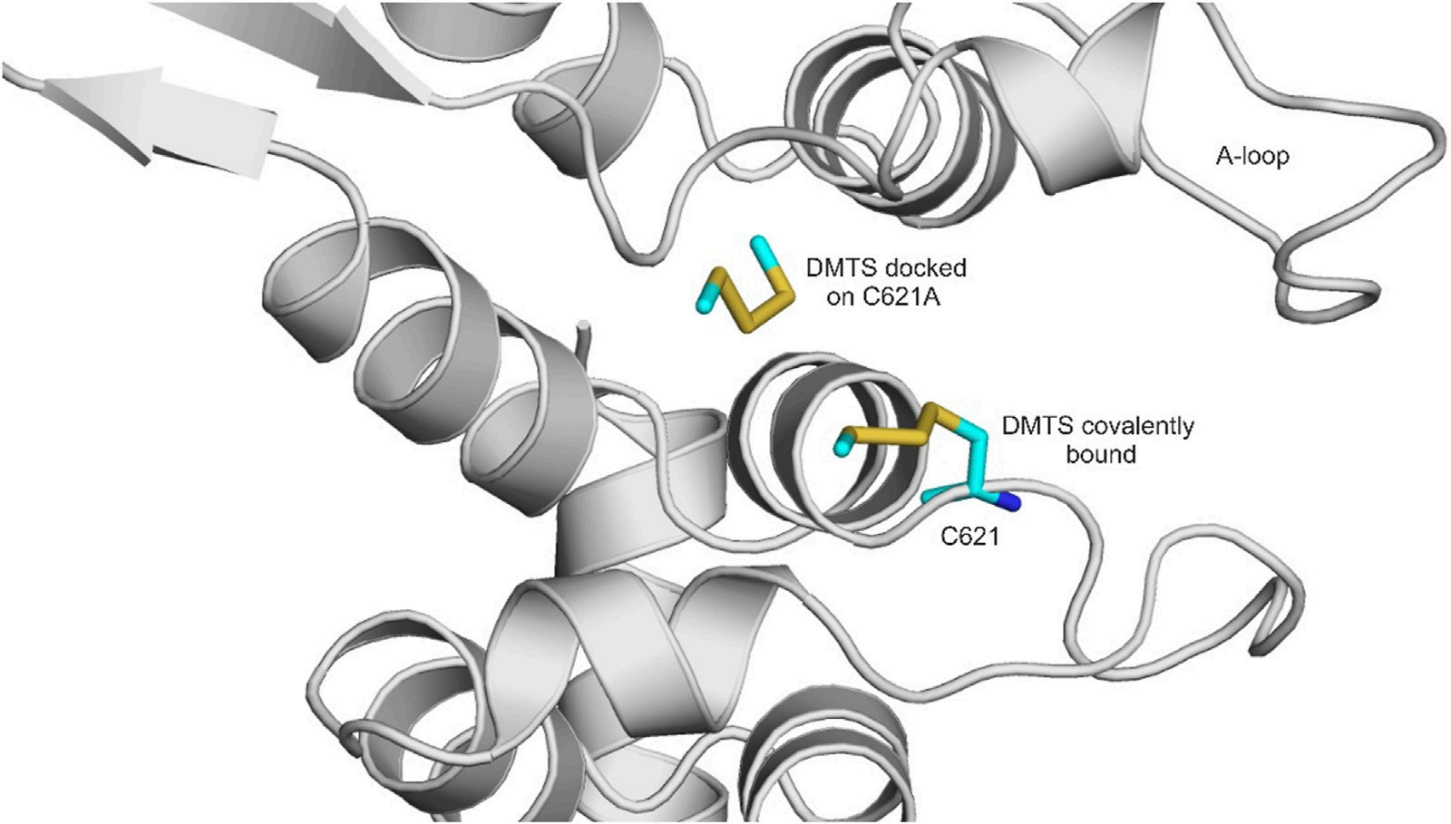
Covalently docked binding position of DMTS to the native holo TRPA1 (attached covalently to C621) and the non-covalently docked binding position of DMTS to the alanine mutant TRPA1 (located further to the left). DMTS is shown as all atom representation sticks (teal). The protein is shown as gray cartoon. A-loop (see Results) is labeled.

DMTS, DADS, and DATS are polysulfide compounds (Figure 3) that presumably form a disulfide bond with previously unknown active site cysteine residues of the TRPA1 receptor (Pozsgai et al., 2019) (Figure 4), which are important in receptor activation. During ligand binding, an intracellular N-terminal loop (A-loop), defined by amino acid residues 666–680, elicits an upward motion (Zhao et al., 2020; Zsidó et al., 2021). In the present study, we found that in the apo structure, this loop overlays the nucleophilic binding cavity, and the amino acids P666 and F669 play a role in the binding of DMTS, DADS, and DATS. These interactions might play an important role in the beginning of the upward motion of the A-loop. In the holo structures, the ligands do not interact with the A-loop (Table 1).

**TABLE 1:** Interacting amino acids in the binding of DMTS to the binding site cysteines and to the alanine mutant receptor. Amino acids within 3.5 Å distance are included.

	C621	C621A	C641	C665
L609	X	X		
F612	X			
K620	X			
C621	X			
P622	X			
I623	X	X		
T624	X			
L638			X	
D639			X	
F640			X	
C641			X	
M642			X	
L643			X	
N659			X	
K661		X	X	
Y662		X	X	X
L663		X		
Q664		X		X
C665				X
P666				X
T684				X

Regarding the calculated free energies of binding (ΔG_calc_, Table 2), it is important to note that from the perspective of receptor activation, only the occurrence of the covalent binding is significant, and not the actual value of binding affinity (Pozsgai et al., 2019). Furthermore, as the closed (ligand-free) conformation of TRPA1 is not appropriate for ligand binding, the prerequisite binding position of DMTS and DATS is far from the S atom of C621 (Supplementary Table S3), and the molecules have to find their way under the A-loop to reach their covalent binding positions. The ΔG_calc_ of all three compounds similarly reflects strong binding to the different cysteine residues (Table 2), with DATS having the largest negative values due to its larger size. As it was expected, a considerable decrease of ΔG_calc_ can be observed in the case of the C621A mutant due to the missing covalent bond (Table 2), if compared with the wild-type holo and the apo receptors (Table 2; Supplementary Table S4). Furthermore, the interaction list in Table 1 reflects that in the case of C621A, and DMTS occupies a binding pocket far from C621. At the same time, approaching C641 and C665 (common interacting residues in Table 1) shows the possibility of the interaction between the DMTS and these cysteines, if C621 is not available for covalent bonding.

**TABLE 2:** Covalent docking of DMTS, DADS, and DATS with FITTED to the three cysteines forming the nucleophilic binding cavity in the intracellular TRPA1 (6pqp holo structure) agonist binding site. Non-covalent docking of the same ligand to the C621A mutant receptor. All calculated binding free energy values are in kcal/mol.

	C621	C641	C665	C621A
DMTS	−44.51	−47.01	−44.56	−34.59
DADS	−44.82	−46.01	−44.63	−38.93
DATS	−49.83	−49.86	−52.22	−46.02

### 3.2 The changes in TRPA1 function due to the mutations

Since the TRPA1 receptor is a non-selective cation channel, its activity is trackable by the intracellular Ca^2+^ concentration changes. For this purpose, we used the Fluo-4 calcium-sensitive fluorescent flow cytometry and radioactive Ca-45 liquid scintillation counting. The whole-cell patch clamp was used to measure the agonist-activated cation current. For most measurement, we used each agonist in 100 µM concentration: organic polysulfides DMTS, DADS, and DATS; AITC and carvacrol; except JT010, which is much more potent than the others, and it was used in 100 nM concentration (Figure 2). The purity of the factory-made DMTS was ≥98%. The purity of our own synthesized DADS was 90% with 10% DATS, and the purity of DATS was 63.5%, with 36.5% DADS as a contaminant. No other contaminant was found in either synthesized compound; only these two compounds, which are chemically and physically very similar and are also contaminants in their natural source, garlic oil, were found. The purity of DADS and DATS was measured by GC-MS (Supplementary Figures S1, 3), and their functional groups were identified by FTIR (Supplementary Figures S2, 4).

Transient transfection was a good method to test the numerous TRPA1 variants much faster than creating stable expressing cell lines before measurements. However, this method needs the cells transfected each time before the experiment, resulting in fluctuating transfection efficiency, and therefore, fluctuating expression levels of TRPA1 regardless of the mutations. The expression level differences have been compensated by normalizing the results to the effect of the non-electrophile agonist carvacrol (Figure 2), which has a different binding site on the TRPA1, and its effect should be unaffected by the mutations we created. Only carvacrol-sensitive TRPA1 variants were adequate for examining the effects of organic polysulfides because they proved to retain other receptor functions.

#### 3.2.1 Transient intracellular calcium concentration changes in mutant TRPA1-expressing cells induced by organic polysulfides

Each mutant TRPA1 variant expressing CHO cells was stained by Fluo-4 calcium-sensitive fluorescent dye, and then the activating effect of agonists was measured by flow cytometry and compared to that of carvacrol.

**FIGURE 5:**
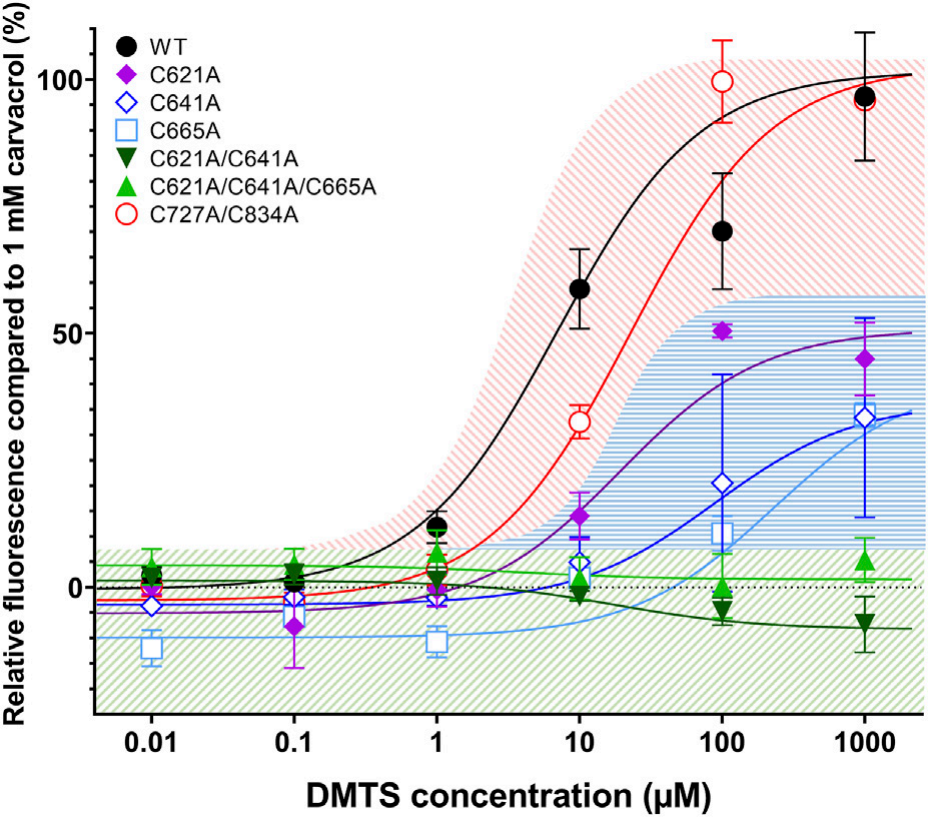
DMTS dose–effect curves in TRPA1 mutant variants, compared to the effect of 1 mM carvacrol. Fluo-4 calcium-sensitive fluorescence was measured by flow cytometry in different TRPA1 variants, using increasing DMTS concentration, compared to the fluorescent signal of 1 mM carvacrol (100%). The single mutation of C621, C641, and C834 decreased the effect of DMTS in TRPA1, but C621A/C641A double mutant and C621A/C641A/C665A triple mutant showed insensitivity to DMTS, indicating that these amino acids are involved in the binding site of the organic polysulfides. The combined double mutation of C727 and C834 (C727A/C834A) made no significant difference from the wild-type TRPA1, suggesting that they are not parts of the binding site of the organic polysulfides. Mean ± SEM, N = 2-4 (number of measurements per TRPA1 variation), n > 10000 (number of cells per measurement).

First, we measured the dose-dependent effect of DMTS on each TRPA1 mutant variant and compared it to that of 1 mM carvacrol (Figure 5). The single mutation of C621, C641, and C665 showed decreased sensitivity to DMTS. Except between C621A and C665A at 100 µM DMTS (*p* = 0.0024), there were no significant differences between the single mutants (*p* > 0.05, two-way ANOVA with Bonferroni multiple comparison test). The C621A/C641A double mutant and C621A/C641A/C665A triple mutant showed insensitivity to DMTS.

In the transmembrane domain, the theoretical binding sites C727 and C834 showed no contribution to the DMTS binding because neither their single (not shown) nor double mutation caused any difference in the effect of DMTS on the TRPA1 receptor, compared to wild-type TRPA1. Therefore, we can reject the idea of C727 and C834 to be parts of the binding site of the strongly hydrophobic organic polysulfides. These mutations were excluded from further experiments.

**FIGURE 6:**
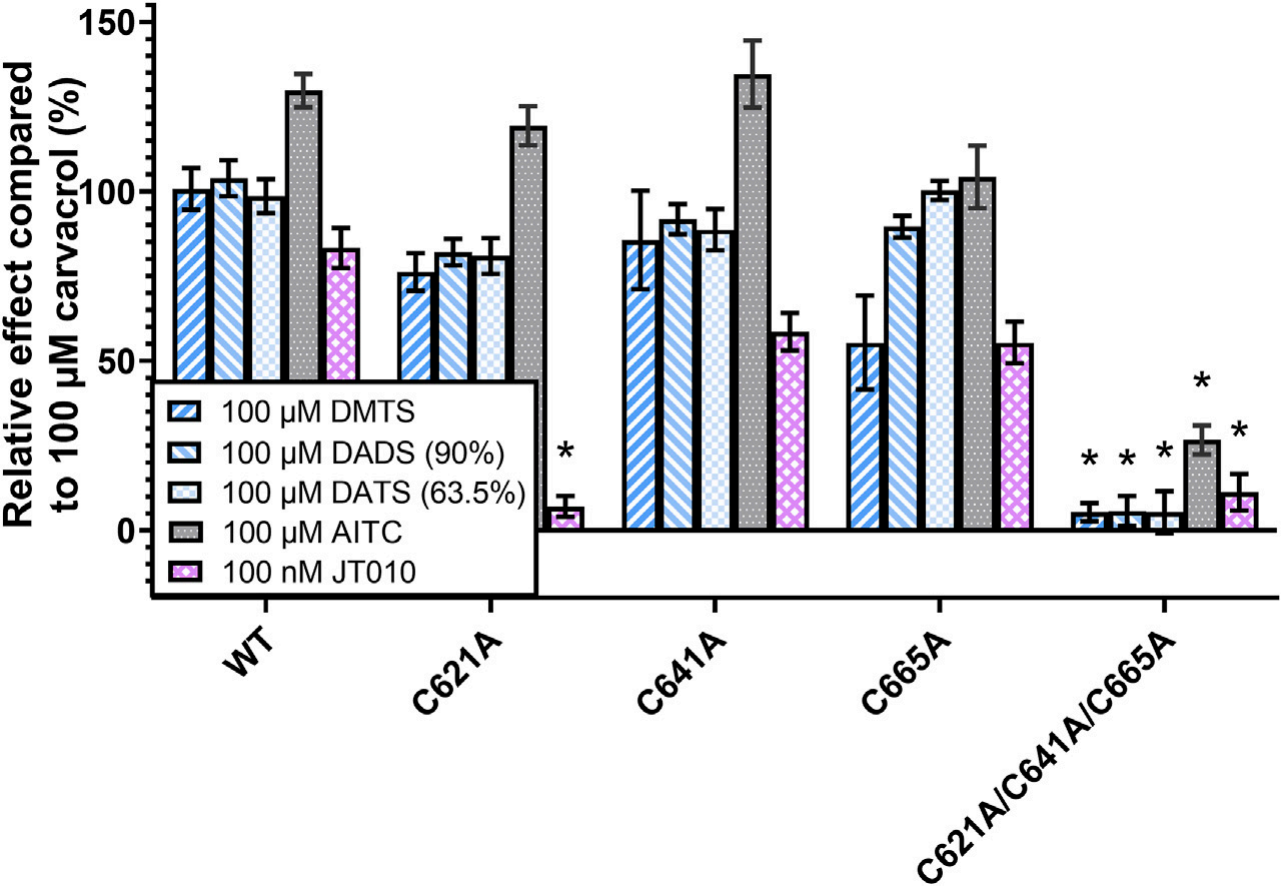
TRPA1 activity measured by calcium-sensitive fluorescence flow cytometry. The different TRPA1 variant-expressing CHO cells were stained by Fluo-4 calcium-sensitive fluorophore. TRPA1 activation causes calcium influx, and therefore increases the intensity of fluorescence, which was measured by flow cytometry. The signal intensity was compared to the effect of 100 µM carvacrol, which was used as a non-electrophilic control agonist. Relatively high concentration (100 µM) of organic polysulfides (DMTS, 90% DADS and 63.5% DATS) showed only slight changes in their receptor-activating effect in the case of TRPA1 single mutants (C621A, C641A, and C665A), but their effect was completely eliminated in the triple-mutant TRPA1 variant (C621A/C641A/C665A). The effect of JT010 was eliminated even in the C621A single mutant, and the other two single mutants also showed a tendency for decreased JT010 sensitivity. Diagram shows the means ± SEM, two-way ANOVA with Bonferroni’s multiple comparison test, significance compared to WT TRPA1: *p < 0.0001, WT TRPA1 replication experiments (N = 4-12), mutant TRPA1 replication experiments (N = 2-5), sample size (*n* = 4–19), cell number per sample (*n* > 2000).

Next, we measured the effect of organic polysulfides DMTS, 90% DADS and 63.5% DATS, as well as AITC and JT010, and compared to that of 100 µM carvacrol (Figure 6). It is important to note that 100 µM carvacrol could not fully activate the TRPA1, in contrast to 1 mM in the previous measurement. This was also the reason why AITC had a consistently higher effect than 100%, except in the triple mutant. The single-mutant TRPA1 variants did not show significant changes, probably due to the limitation of Fluo-4 dye, which was easily saturated by calcium at the relatively high organic polysulfide concentration (100 µM). However, C621A had completely lost sensitivity to JT010, which is strong evidence that C621 binds to this agonist covalently. JT010 showed decreased efficacy in C641A and C665A mutations, which suggests their assisting role in binding JT010 to C621. The triple mutation of C621, C641, and C665 resulted in complete insensitivity to DMTS, DATS, DADS, and JT010, but only minimized the effect of AITC. There was no significant difference between the effects of the three organic polysulfides.

#### 3.2.2 Radioactive Ca-45 uptake in mutant TRPA1-expressing cells induced by organic polysulfides

**FIGURE 7:**
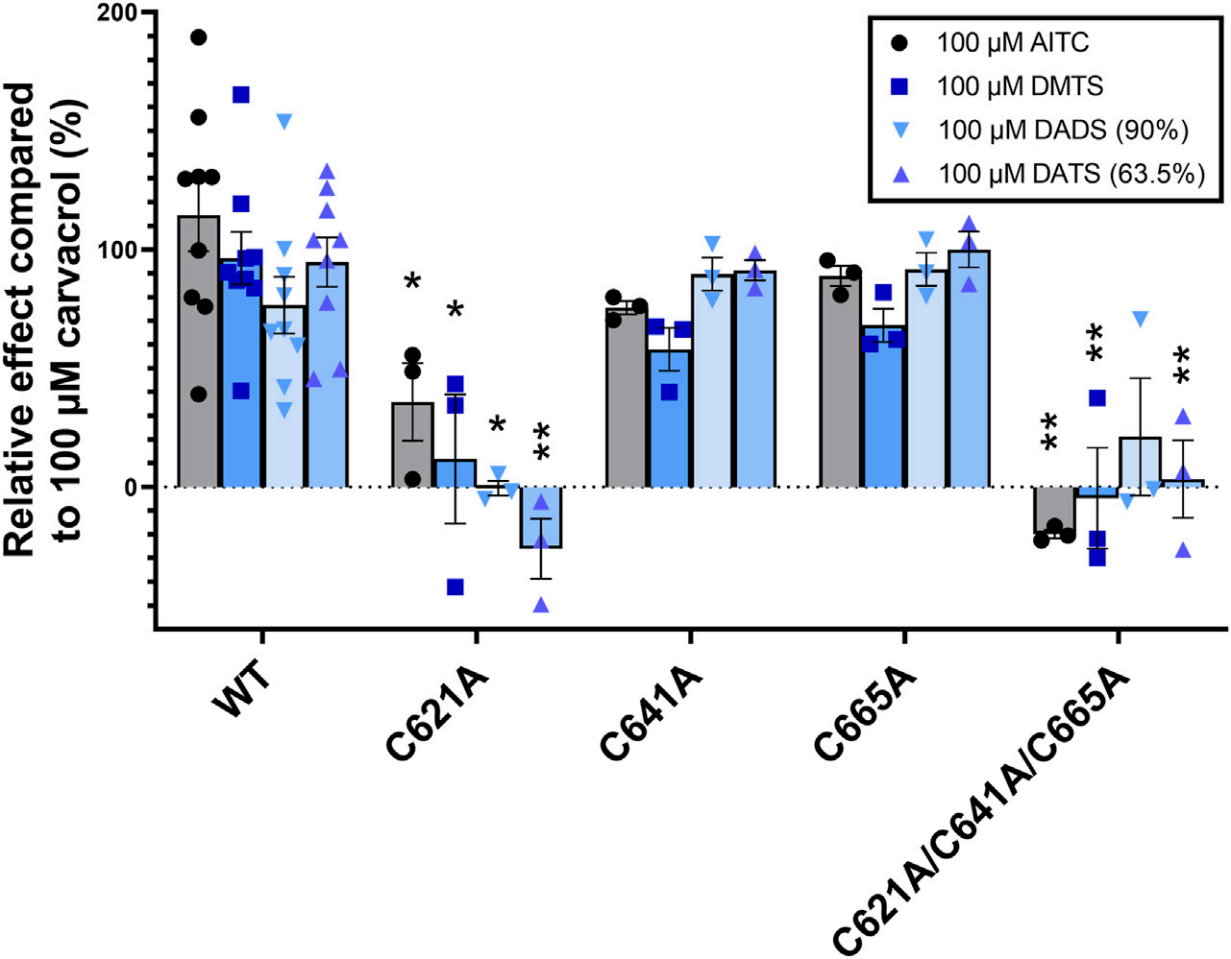
TRPA1 activity measured by radioactive Ca-45 liquid scintillation counting. The radioactive Ca-45 uptake due to TRPA1 activation was measured by liquid scintillation counting and compared to the effect of 100 µM carvacrol. Out of the three TRPA1 single mutants, C612A shows the highest decrease in sensitivity to electrophilic agonists, indicating its key role in agonist binding. Triple mutation eliminated the effect of electrophilic agonists. Diagram shows the individual values and means ± SEM, two-way ANOVA with Bonferroni’s multiple comparison test, significance compared to WT TRPA1: *p < 0.005, **p < 0.0001, *n* = 3–6

To confirm the results with an alternative method and to better understand the priorities between C621, C641, and C665 cysteines in binding the organic polysulfides, we tested the effect of DMTS, 90% DADS, 63.5% DATS, and AITC on each mutant TRPA1 variant in the radioactive Ca-45 uptake assay (Figure 7). The radioactive Ca-45 uptake due to TRPA1 activation was measured by liquid scintillation counting. The results were compared to those of 100 µM carvacrol. C641A and C665A single mutations showed no significant changes, suggesting the secondary role of these cysteines in binding organic polysulfides. C621A and the triple mutant had significantly reduced sensitivity to all measured electrophilic agonists, except for the triple mutant to DADS due to an outlier. There was no significant difference between the effects of the three organic polysulfides.

#### 3.2.3 Current-activating effect of organic polysulfides in mutant TRPA1-expressing cells

**FIGURE 8:**
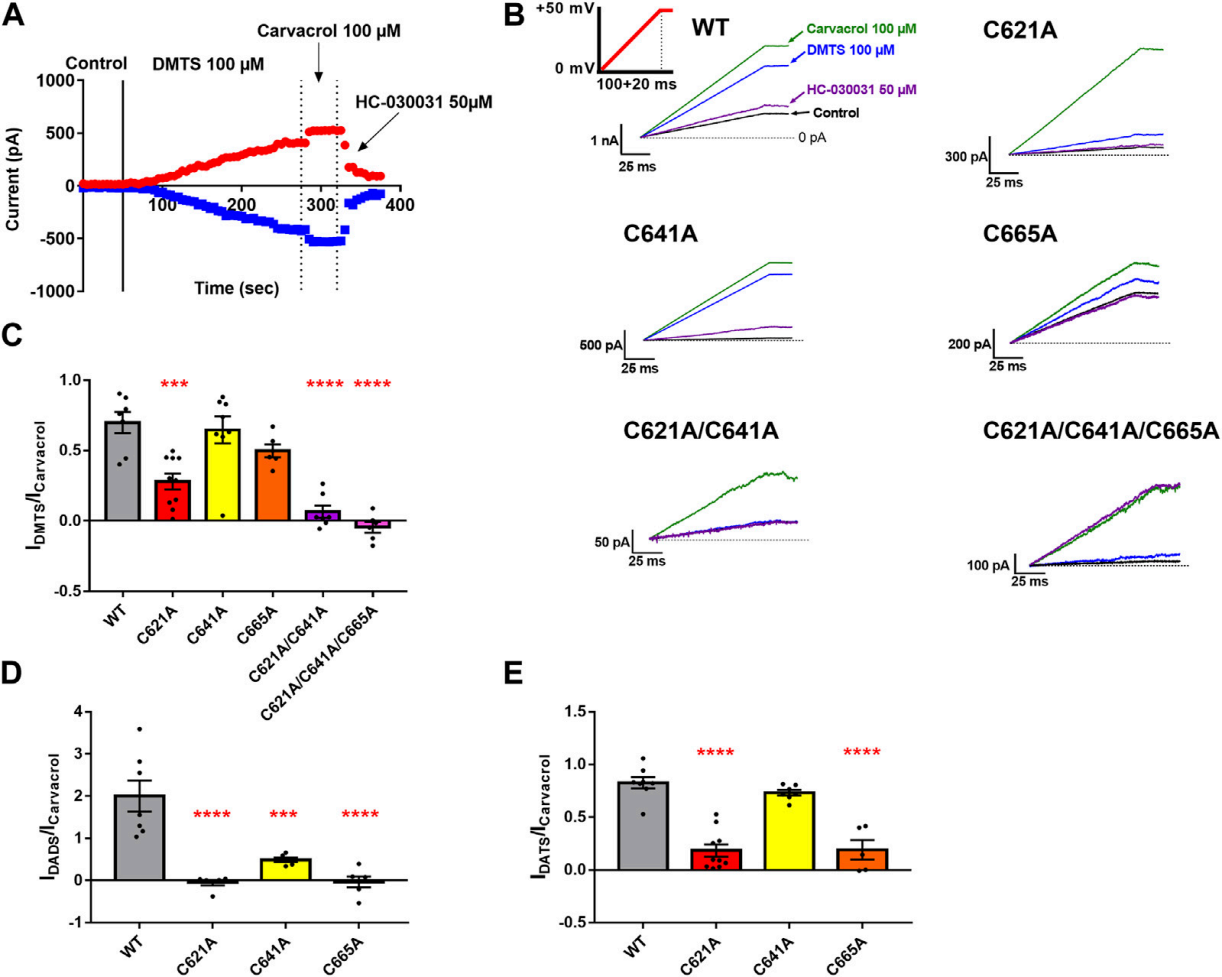
Effect of the polysulfides on the WT and on the cysteine mutant TRPA1 using the whole -cell patch-clamp technique. **(A)** Representative whole-cell patch-clamp measurement of a WT TRPA1-expressing CHO cell. Until the continuous line, Ca2+ free control solution was applied, and between the continuous and the first dotted line, the effect of 100 µM DMTS is shown. Between the two dotted lines, and after the second dotted line, we can see the effects of the two positive controls (carvacrol and HC-030031, respectively). The red symbols represent the peak currents at +50 mV, while the blue ones are the peak currents at -50 mV command voltage. **(B)** Representative whole-cell measurements regarding the WT, C621A, C641A, C665A, C621A/C641A, and C621A/C641A/C665A populations (saturated traces). The green, blue, purple, and black solid lines were measured in the presence of 100 µM carvacrol, 100 µM DMTS, 50 µM HC-030031, and Ca2+-free control solution, respectively. The horizontal dashed line represents 0 pA current. The voltage protocol is shown with a solid red line as an inset on the top left panel. The fluctuation of the effect of carvacrol correlated to and indicated the overall TRPA1 response rate. **(C–E)** Bar charts of the activated current ratios by the application of 100 µM DMTS, 90% DADS, and 63.5% DATS, respectively. The colored bars represent the WT and the C621A, C641A, C665A, C621A/C641A, and C621A/C641A/C665A mutant TRPA1 (N_transfection_≥2 and n_cell_≥5 for each population). Black dots indicate individual measurements, and bar chart height and error bars show mean ± SEM. Asterisks represent significant difference (*p ≤ 0.05, **p ≤ 0.01, ***p ≤ 0.001, and ****p ≤ 0.0001).

To further confirm the possible binding sites of the tested sulfide-containing agonists, we used the patch-clamp technique and determined the current-activating ability of DMTS, 90% DADS, and 63.5% DATS relative to carvacrol. Using the formula described in the Materials and methods section, a ratio was calculated, which shows the relative channel-opening ability of the tested agonists normalized to the current-activating effect of the known non-electrophilic agonist carvacrol. Figures 8A,B show the representative measurements of DMTS, while in Figures 8C–E, the current activation ratios are plotted. Mutating C621 to alanine significantly decreased the effect of the polysulfides compared to the WT. The mutation in C665 also affected the current-activating ability of DADS and DATS, but failed to significantly alter the effect of DMTS. Mutation C641A did not influence the agonist effect of DMTS and DATS, but only modified the effect of DADS. We also have checked the effect of DMTS on the double (C621A/C641A) and triple (C621A/C641A/C665A) mutants (Figures 8B, C). The TRPA1 currents of the double- and triple-mutant channels in the presence of DMTS were significantly reduced compared to the WT (*p* ≤ 0.0001 for both) and also compared to the C621A mutant (*p* = 0.01 and 0.0006, respectively, data not shown). DADS showed a stronger effect on WT TRPA1 than the other organic polysulfides, compared to 100 µM carvacrol. The C621A/C641A/C665A triple mutant showed insensitivity to HC-030031 antagonist.

## 4 Discussion

The activating effect of garlic-derived organic polysulfides on the TRPA1 receptor is well-established (Koizumi et al., 2009; Terada et al., 2014; Pozsgai et al., 2017; 2019; Bátai et al., 2018; 2022; Dombi et al., 2021; Mahajan et al., 2021), but we are the first to create site-directed mutant variants of TRPA1 to identify the binding site of organic polysulfides. Our data were acquired on these mutant TRPA1 variants by *in silico* modeling and three different functional methods: whole-cell patch clamp, radioactive calcium-45 liquid scintillation counting, and calcium-sensitive fluorescent flow cytometry. The results are overlapping and strongly support each other. In the present study, we have proven that organic polysulfides DMTS, DADS, and DATS bind covalently to the C621, C641, and C665 amino acids activating the human TRPA1 receptor, respectively. C621 had the most important role, but C641 and C665 also contributed to binding the electrophilic agonists. Only their combined triple mutation led the TRPA1 to become completely insensitive to organic polysulfides.

Out of the three polysulfides, DATS, the largest ligand, showed the most favorable calculated free energy of binding; however, as it was highlighted by experimental results, the formation of a covalent bond with C621 is more important than the strength of the binding interactions. Notably, the apo TRPA1 conformation is not favorable for binding the electrophilic agonists, as seen in the prerequisite docking calculations. Prerequisite docking on the holo target protein resulted in smaller distances between the covalently binding atoms. This observation is explained by the overlapping A-loop in the binding site that hinders ligand accessibility in the apo structure, but not in the holo structure. As shown in our previous paper (Zhao et al., 2020; Zsidó et al., 2021), the upward motion of the A-loop after the prerequisite binding of the agonist to P666 and F669 is necessary for the accessibility of the actual binding site.

These three cysteine residues have been known to conform the binding site of most electrophilic agonists, with the exception of AITC, which additionally binds to the K710 lysine, and the irreversible agonist JT010, whose activating effect can be abolished entirely by the mutation of C621 alone (Hinman et al., 2006; Deering-Rice et al., 2011; Eberhardt et al., 2012; Shapiro et al., 2013; Suo et al., 2020).

In contrast to the patch clamp and calcium-45 liquid scintillation measurements, the calcium-sensitive fluorescent flow cytometry did not show distinction between the single-mutant TRPA1 variants. This was probably due to the limitation of Fluo-4 dye, which could be easily saturated by calcium at the relatively high concentration (100 µM) of organic polysulfides. Therefore, radioactive calcium-45 liquid scintillation counting and whole-cell patch clamp are more precise and more reliable methods, and we could use them to find the differences between the three cysteine residues.

Out of these three cysteine residues, the key role of C621 in organic polysulfide binding has been predicted by our *in silico* model and supported by the results of calcium-45 liquid scintillation counting and whole-cell patch clamp. The key role of C621 is well-known for numerous electrophilic agonists, such as JT010, iodoacetamide, BODIPY-iodoacetamide, AITC, N-benzylthioformamide (9BE), and benzyl isothiocyanate (Macpherson et al., 2007; Eberhardt et al., 2012; Bahia et al., 2016; Suo et al., 2020; Habgood et al., 2022). Whole-cell patch clamp showed C665 as the second-most important cysteine in organic polysulfide binding. The secondary role of C665 has also been shown in cases of some other electrophilic agonists, such as iodoacetamide, BODIPY-iodoacetamide, N-ethylmaleimide, and benzyl isothiocyanate (Wang et al., 2004; Bahia et al., 2016; Suo et al., 2020; Habgood et al., 2022).

Although a single mutation of either cysteine only reduces the efficacy of organic polysulfides, the activating effect of JT010 was completely eliminated by the single mutation of C621. Several pieces of evidence have shown that JT010 only binds to C621 covalently, and not to other cysteines (Takaya et al., 2015; Suo et al., 2020; Matsubara et al., 2022). According to this fact, the reduced effect of JT010 on TRPA1 with the single mutation of C641 or C665 suggests other roles of these cysteines instead of directly binding the electrophilic agonist, which has been shown in our calcium-sensitive fluorescent flow cytometry. Presumably, C641 and C665 amino acids maintain the structure of the binding pocket or create an attracting environment for the electrophilic agonists or both. Since the single mutation of C621 was not enough for the complete inhibition of polysulfides, we conclude that C641 and C665 also bind covalently to these compounds. Only the combined triple mutation of these cysteines completely abolished the activating effect of organic polysulfides. Other electrophilic agonists have also been reported to lose effects on the triple cysteine mutant TRPA1 variant, except for the AITC, which retains a minimal effect in the presence of intact K710 lysine (Hinman et al., 2006; Deering-Rice et al., 2011; Eberhardt et al., 2012; Shapiro et al., 2013). According to our calcium-sensitive fluorescent flow cytometry data, AITC showed higher efficacy than the control agonist non-electrophilic carvacrol both in wild-type TRPA1 and single-mutant variants. In contrast with the total insensitivity to the organic polysulfides, triple-mutant TRPA1 showed a minimal sensitivity to AITC.

Neither the single nor the combined double mutation of C727 and C834 made any difference in the effect of DMTS, compared to wild-type TRPA1. Therefore, we have to reject the idea that C727 and C834 are parts of the binding site for highly hydrophobic organic polysulfides, and possibly other electrophilic agonists.

In HEK293 cells expressing mouse Trpa1, C415, and C422, cysteine residues have been shown to bind inorganic polysulfide (disodium trisulfide) and other electrophilic agonists (Macpherson et al., 2007; Fujita et al., 2008; Hatakeyama et al., 2015). Whether the human homologous cysteine residues (C414 and C421) or the remaining 21 out of 28 cysteine residues have any role in binding organic polysulfides and other electrophilic agonists has to be clarified.

Out of the organic polysulfides we used, the factory-made DMTS was almost pure (≥98%), but the synthesized DADS and DATS were inseparable, as our DADS was 90% and the DATS was 63.5%, accompanying each other much like in their natural source, garlic oil. The whole-cell patch clamp showed the effect of DADS to be higher on WT TRPA1, but more affected by single mutations than the effect of other organic polysulfides. C621A and C665A single mutants showed decreased sensitivity to DMTS and DATS, and they were completely insensitive to DADS. The C641A single mutant showed decreased sensitivity to DADS, but not to DMTS and DATS. This finding may suggest a higher efficacy of DADS, but this needs to be further investigated as it is not supported by our data from other measurement methods, in which the three organic polysulfides showed equivalent effects. In the whole-cell patch clamp technique, the C621A/C641A/C665A triple mutant showed insensitivity to antagonist HC-030031, which needs further investigation. The triple-mutant receptors were completely insensitive to polysulfides, but their remained functional ability was proven by the fact that they still showed well-measurable activity in response to carvacrol.

We have to mention that the main limitation of the present study is that the overall TRPA1 response rate fluctuated from transfection to transfection in cell lines, which was the drawback of the applied transient transfection method. The transient transfection efficiency is highly dependent on a wide range of conditions, and even slight changes may lead to different results, and therefore, practically, it has intrinsic variability. The complexity of expression from transiently transfected plasmids highlights the importance of appropriate experimental controls (Bollin et al., 2011; Nejepinska et al., 2012; Di Blasi et al., 2021; Fus-Kujawa et al., 2021). The fluctuation of the effect of carvacrol correlated to and indicated the overall TRPA1 response rate. After a detailed analysis of our data, we are confident that the sensitivity of the TRPA1 variants did not change in response to carvacrol. This has been demonstrated by the consistent [electrophilic agonist/carvacrol] effect ratio in our repeated experiments. For the equivalent C621S/C641S/C665S triple-mutant human TRPA1, carvacrol has been used commonly as a positive control agonist and shown to retain its complete effect (Babes et al., 2013; Ibarra and Blair, 2013; Meents et al., 2016; Schenk et al., 2019).

Using three functional tests, we have proven that organic polysulfides covalently bind to C621, C641, and C665 cysteine residues activating the TRPA1 receptor, and only their combined triple mutation is able to abolish the effect of organic polysulfides. We also identified C621 as the most important and C665 as the second most important cysteine in the binding of organic polysulfides. This highly overlaps with the binding site of most electrophilic agonists on the TRPA1 receptor, which, in turn, may further clarify the binding mechanism of the other electrophilic agonists.

Our present study has not only demonstrated that TRPA1 can be an important target molecule for organic polysulfides but also determined their precise binding sites using mutant receptors.

## Figures and Tables

**Table:** 

**AITC**	Ally isothiocyanate (mustard oil)
**Amp**	Ampicillin, an antibiotic used for selective bacterium growth
**BODIPY-iodoacetamide**	4,4-Difluoro-5,7-dimethyl-4-bora-3a,4a-diaza-s-indacene-3-yl)methyl)iodoacetamide, a fluorescent dye-conjugated iodoacetamide
**C414**	Cysteine in the 424th position in the human TRPA1 peptide sequence
**C415**	Cysteine in the 415th position in the mouse Trpa1 peptide sequence
**C421**	Cysteine in the 421st position in the human TRPA1 peptide sequence
**C422**	Cysteine in the 422nd position in the mouse Trpa1 peptide sequence
**C621**	Cysteine in the 621st position in the human TRPA1 peptide sequence
**C621A**	Mutation, in which the cysteine is replaced by alanine in the 621st position in the human TRPA1 peptide sequence
**C621S**	Mutation, in which the cysteine is replaced by serine in the 621st position in the human TRPA1 peptide sequence
**C641**	Cysteine in the 641st position in the human TRPA1 peptide sequence
**C641A**	Mutation, in which the cysteine is replaced by alanine in the 641st position in the human TRPA1 peptide sequence
**C641S**	Mutation, in which the cysteine is replaced by serine in the 641st position in the human TRPA1 peptide sequence
**C665**	Cysteine in the 665th position in the human TRPA1 peptide sequence
**C665A**	Mutation, in which the cysteine is replaced by alanine in the 665th position in the human TRPA1 peptide sequence
**C665S**	Mutation, in which the cysteine is replaced by serine in the 665th position in the human TRPA1 peptide sequence
**C727**	Cysteine in the 727th position in the human TRPA1 peptide sequence
**C834**	Cysteine in the 834th position in the human TRPA1 peptide sequence
**CHO**	Chinese hamster ovary cells, mammalian model cells excellent for recombinant protein expression
**DADS**	Diallyl disulfide
**DATS**	Diallyl trisulfide
**DMEM**	Dulbecco’s modified Eagle’s medium, a minimal essential medium for mammalian cell growth
**DMSO**	Dimethyl sulfoxide, an organic solvent miscible with both polar and apolar compounds
**DMTS**	Dimethyl trisulfide
**GC-MS**	Gas chromatography mass spectrometry
**GFP**	Green fluorescent protein, a reporter protein
**HEK293 cells**	Human embryotic kidney 293 cells, human model cells excellent for human protein expression
**JT010**	2-Chloro-*N*-[4-(4-methoxyphenyl)-2-thiazolyl]-*N*-(3-methoxypropyl)acetamide
**K710**	Lysine in the 710th position in the human TRPA1 peptide sequence
**LB medium**	Lysogeny broth medium, a simple medium for bacterium growth
**PCR**	Polymerase chain reaction, a DNA fragment amplifying technique
**SDS**	Sodium dodecyl sulfate, a strong organic detergent used for lysing cells
**TRPA1**	Human transient receptor potential ankyrin 1 receptor
**Trpa1**	Mouse transient receptor potential ankyrin 1 receptor
**WT**	Wild type, not mutated, intact (gene; protein)

## Data Availability

The original contributions presented in the study are included in the article/Supplementary Material; further inquiries can be directed to the corresponding authors.
