# Supplementary material for: Elucidation of the binding mode of organic polysulfides on the human TRPA1 receptor
Source: Front Physiol. 2023 Jun 7;14:1180896. doi: 10.3389/fphys.2023.1180896 (PMC10282659; doi:10.3389/fphys.2023.1180896)
Supplement: Supplementary file 1 [file DataSheet1.docx]

Supplementary Material

# PCR programs

| **Step** | | **Temperature (°C)** | **Time** |
| --- | --- | --- | --- |
| Initial denaturation | | 98 | 5 min |
| 5 cycles | denaturation | 98 | 30 s |
|  | annealing | 40 | 30 s |
|  | extension | 72 | 90 s |
| 5 cycles | denaturation | 98 | 30 s |
|  | annealing | 45 | 30 s |
|  | extension | 72 | 90 s |
| 30 cycles | denaturation | 98 | 30 s |
|  | annealing | 50 | 30 s |
|  | extension | 72 | 90 s |
| Final extension | | 72 | 10 min |

**Supplementary Table 1A.** First PCR program of site-directed mutagenesis

The annealing temperature starts low because of the initial mismatches, but the gradual increase leads to optimal site specificity.

| **Step** | | **Temperature (°C)** | **Time** |
| --- | --- | --- | --- |
| Initial denaturation | | 98 | 5 min |
| 5 cycles | denaturation | 98 | 30 s |
|  | annealing | 50 | 30 s |
|  | extension | 72 | 120 s |
| 30 cycles | denaturation | 98 | 30 s |
|  | annealing | 55 | 30 s |
|  | extension | 72 | 120 s |
| Final extension | | 72 | 10 min |

**Supplementary Table 1B.** Second PCR program of site-directed mutagenesis

# PCR primers

| **Name** | **Sequence** | **length (bp)** | **GC content (%)** | **Melting temperature (°C)** |
| --- | --- | --- | --- | --- |
| **Sequencing primers** | | | | |
| CysF | CAG TGA CCA CAA TGG CTG GAC | 21 | 57.1 | 58.5 |
| CysR | GCA TCA TGC TGA AGG TCT GG | 20 | 55.0 | 56.2 |
| **Cloning primers** | | | | |
| CysF2 | GTC AGA GGC TCC TAC AAG ACA | 21 | 52.4 | 55.9 |
| CysR2 | TGA AGT TCC ACC TGC ATA GC | 20 | 50.0 | 55.0 |
| **Mutation primers** | | | | |
| CYS621F (C621A-F) | CAG GCA ATA AAG CTC CAA TTA CAG A | 25 | 40.0 | 55.4 |
| CYS621R (C621A-R) | TCT GTA ATT GGA GCT TTA TTG CCT G | 25 | 40.0 | 55.4 |
| CYS641F (C641A-F) | TTT TAG ATT TCG CTA TGT TGC ATT C | 25 | 32.0 | 52.5 |
| CYS641R (C641A-R) | GAA TGC AAC ATA GCG AAA TCT AAA A | 25 | 32.0 | 52.5 |
| CYS665F (C665A-F) | AAT ATC TTC AAG CTC CAT TAG AAT T | 25 | 28.0 | 50.5 |
| CYS665R (C665A-R) | AAT TCT AAT GGA GCT TGA AGA TAT T | 25 | 28.0 | 50.5 |
| CYS727F (C727A-F) | TAG GAT CTT ACG CTC TTG GTC TCA T | 25 | 44.0 | 56.9 |
| CYS727R (C727A-R) | ATG AGA CCA AGA GCG TAA GAT CCT A | 25 | 44.0 | 56.9 |
| CYS834F (C834A-F) | TGC AGT GGC AAG CTG GAG CAA TTG C | 25 | 56.0 | 64.6 |
| CYS834R (C834A-R) | GCA ATT GCT CCA GCT TGC CAC TGC A | 25 | 56.0 | 64.6 |

**Supplementary Table 2.** Primers used in site-directed mutagenesis and for control sequencing

# Supplementary Methods

Prerequisite binding calculations were performed by AutoDock (RRID: SCR_012746 (Morris et al., 2009)). The number of grid points was set to 60×60×60 with a 0.375 Å grid spacing. Lamarckian genetic algorithm was used, flexibility on all active torsions were allowed on the ligands. Ten docking runs were performed for all ligands, and the resulting ligand conformations were ranked based on their ΔG_calc_ values.

# Supplementary Results

*Binding mode of DMTS and DATS to the C621S mutant receptor*. In the mutant receptor structure, the active site cysteine (C621) is mutated to a serine amino acid. In this case a disulfide bond cannot be formed between DMTS or DATS and the receptor, and the covalent interaction does not occur. (Suo et al., 2020) showed, that in the case of the C621S mutant, administration of JT010 or 9BE does not exhibit TRPA1 currents, however if only another active site cysteine is mutated (C665S) the response is still held and a robust current is observed. This leads to the conclusion, that in the absence of the C621 cysteine, even if the agonists bound to another nearby cysteine the receptor activation would not occur in the case of non-polysulfide compounds. In the case of the C621S receptor, DMTS binds non-covalently with a significantly lower ΔG_calc_ (to the same binding pocket), than to the wild type receptor (**Table S3a**). Interestingly, despite DATS also binds non-covalently to the agonist binding pocket of TPRA1, its ΔG_calc_ is similar, to that observed in the case of the native receptor.

| Ligand name | DMTS | DATS |
| --- | --- | --- |
| ΔG_calc_ (kcal/mol) | -2.32 | -2.98 |
| Rank | 1/3 | 1/3 |
| Covalently binding atom distance from CYS621 S atom (Å) | 7.2 | 10.8 |

**Supplementary Table 3A.** Prerequisite docking calculations of TRPA1 agonists performed by AutoDock 4.2 (Morris et al., 1998) on the native apo structure (6v9w)

| Ligand name | DMTS | DATS |
| --- | --- | --- |
| ΔG_calc_ (kcal/mol) | -2.43 | -3.26 |
| Rank | 2/2 | 1/2 |
| Covalently binding atom distance from CYS621 S atom (Å) | 5.2 | 5.4 |

**Supplementary Table 3B.** Prerequisite docking calculations of TRPA1 agonists performed by AutoDock 4.2 (Morris et al., 1998) on the native holo structure (6pqp)

| Ligand name | DMTS | DATS |
| --- | --- | --- |
| ΔG_calc_ (kcal/mol) | -28.788 | -35.36 |
| Rank | 1/10 | 1/10 |
| Covalently binding atom distance from CYS621 S atom (Å) | 3.7 | 3.7 |

**Supplementary Table 3C.** Prerequisite docking calculations of TRPA1 agonists performed by FITTED on the native holo structure (6pqp)

| Ligand name | DMTS | DATS |
| --- | --- | --- |
|  | APO | |
| ΔG_calc_ (kcal/mol) | -45.766 | -45.393 |
| Rank | 1/5 | 1/5 |
| Covalent bond type | disulfide | disulfide |
|  | C621S mutant | |
| ΔG_calc_ (kcal/mol) | -35.8 | -46.435 |
| Rank | 1/5 | 1/5 |
| Covalent bond type | non-covalent | non-covalent |
|  | C621A mutant | |
| ΔG_calc_ (kcal/mol) | -34.59 | -46.02 |
| Rank | 1/3 | 1/3 |
| Covalent bond type | non-covalent | non-covalent |

**Supplementary Table 4A.** Non-covalent docking calculations of DMTS and DATS to the apo and mutant TRPA1 receptors performed by FITTED

| Ligand name | DMTS | DATS |
| --- | --- | --- |
| ΔG_calc_ (kcal/mol) | -44.641 | -50.167 |
| Rank | 1/10 | 1/10 |
| Covalent bond type | disulfide | disulfide |

**Supplementary Table 4B.** Covalent docking calculations of TRPA1 agonists to C621 performed by FITTED on the native holo structure (6pqp)


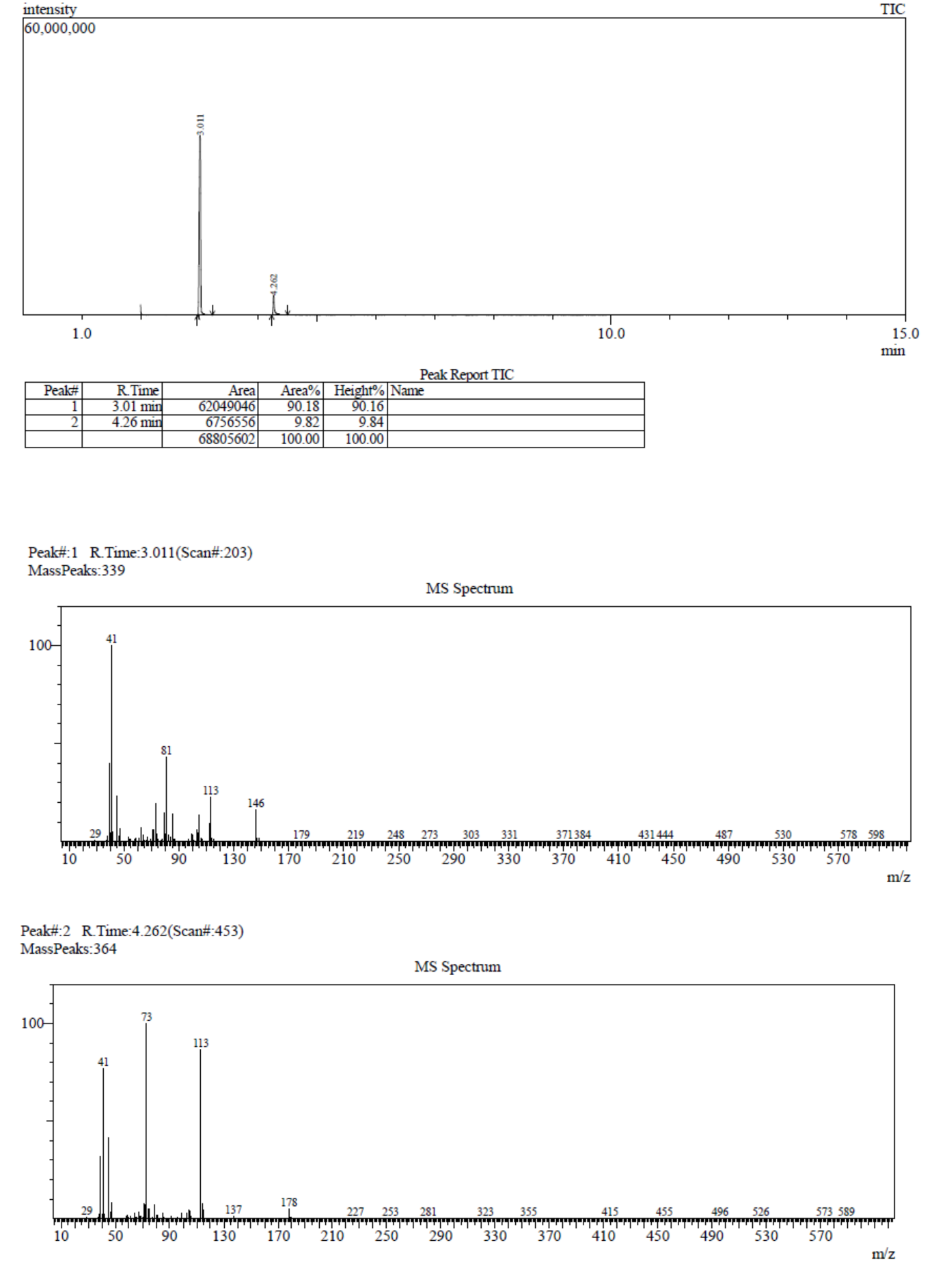


**Supplementary Figure 1.** GC-MS of diallyl disulfide

Peak Report TIC

| Peak# | R. Time | Area | Area% | Height% | Name |
| --- | --- | --- | --- | --- | --- |
| 1 | 3.01 min | 62049046 | 90.18 | 90.16 | DADS |
| 2 | 4.26 min | 6756556 | 9.82 | 9.84 | DATS |
|  |  | 68805602 | 100.00 | 100.00 |  |

**Supplementary Figure 2.** FT-IR of diallyl disulfide


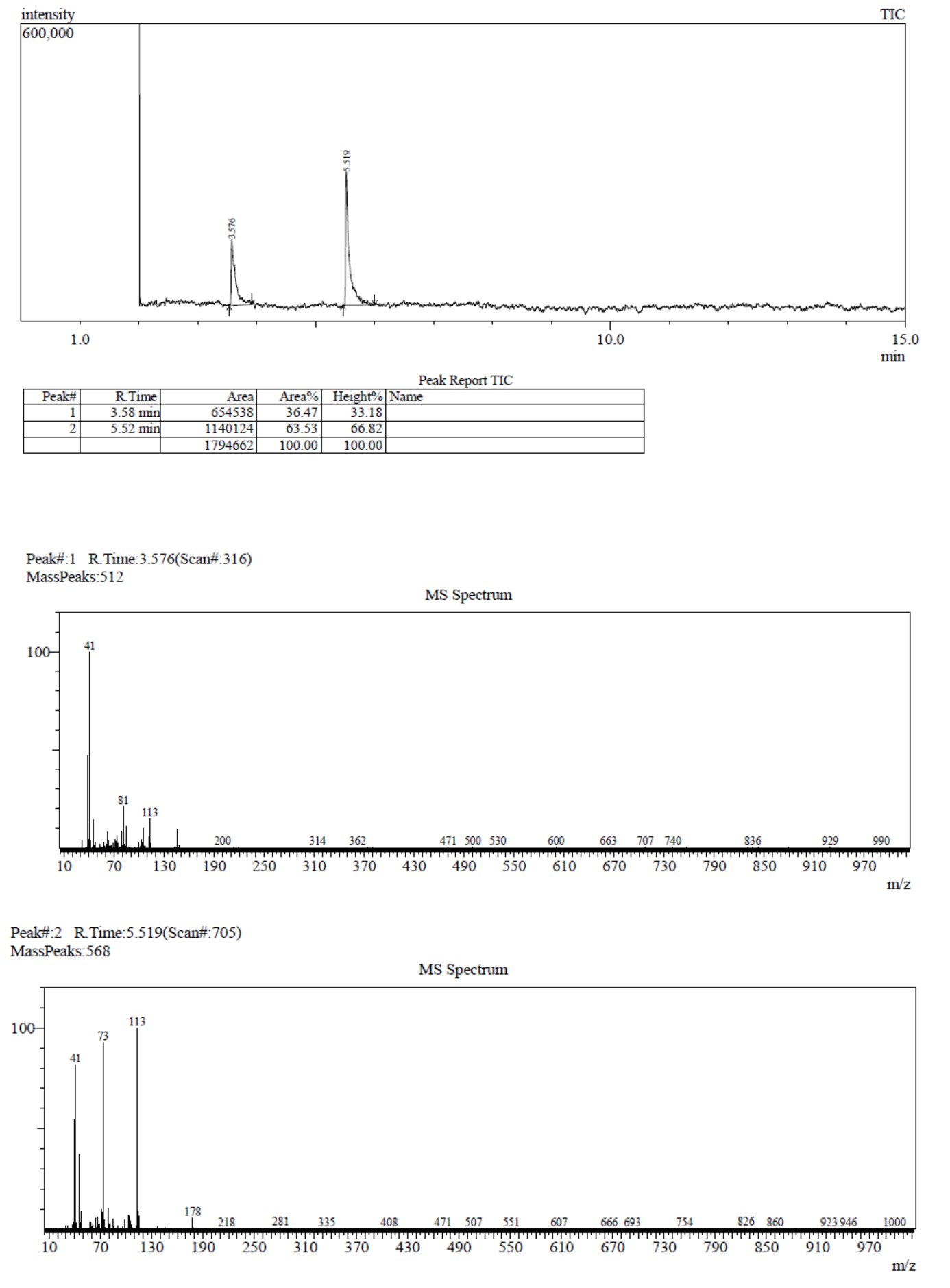


**Supplementary Figure 3.** GC-MS of diallyl trisulfide

Peak Report TIC

| Peak# | R. Time | Area | Area% | Height% | Name |
| --- | --- | --- | --- | --- | --- |
| 1 | 3.58 min | 654538 | 36.47 | 33.18 | DADS |
| 2 | 5.52 min | 1140124 | 63.53 | 66.82 | DATS |
|  |  | 1794662 | 100.00 | 100.00 |  |

**Supplementary Figure 4.** FT-IR of diallyl trisulfide

# References

Morris, G. M., Goodsell, D. S., Halliday, R. S., Huey, R., Hart, W. E., Belew, R. K., et al. (1998). Automated docking using a Lamarckian Genetic Algorithm and empirical binding free energy function. *Journal of Computational Chemistry* 19, 1639–1662.

Suo, Y., Wang, Z., Zubcevic, L., Hsu, A. L., He, Q., Borgnia, M. J., et al. (2020). Structural Insights into Electrophile Irritant Sensing by the Human TRPA1 Channel. *Neuron* 105, 882-894.e5. doi: 10.1016/j.neuron.2019.11.023.
